# Viroid‐derived small RNA induces early flowering in tomato plants by RNA silencing

**DOI:** 10.1111/mpp.12721

**Published:** 2018-09-28

**Authors:** Charith Raj Adkar‐Purushothama, Teruo Sano, Jean‐Pierre Perreault

**Affiliations:** ^1^ RNA Group/Groupe ARN, Département de Biochimie Faculté de Médecine des Sciences de la Santé Pavillon de Recherche Appliquée au Cancer Université de Sherbrooke 3201 rue Jean­Mignault Sherbrooke QC J1E 4K8 Canada; ^2^ MYM Nutraceuticals Inc 1500 ‐ 409 Granville Street Vancouver BC V6C 1T2 Canada; ^3^ Faculty of Agriculture and Life Science Hirosaki University Hirosaki 036‐8561 Japan

**Keywords:** early flowering, FRIGIDA‐like protein 3, host–pathogen interaction, PSTVd, RNA silencing, vd‐sRNA, viroid.

## Abstract

Viroid infection often leads to early flowering in the host plant. This report describes the targeting of the *FRIGIDA‐like protein 3* (*FRL3*) mRNA in tomato plants by a small RNA derived from the conserved left terminal region of the potato spindle tuber viroid (PSTVd). This targeting leads to the silencing of the *FRL3* mRNA. Viroid infection assays using a severe variant of PSTVd induced early flowering in tomato plants by the down‐regulation of greater amounts of the target than did a mild PSTVd variant. The targeting of the *FRL3* mRNA by RNA silencing was validated by both an artificial microRNA experiment transiently expressing viroid‐derived small RNAs in tomato plants, and by 5′ RNA ligase‐mediated rapid amplification of cDNA ends (RACE). These data unambiguously demonstrated the role of small RNAs in the early flowering seen in viroid‐infected plants.

## INTRODUCTION

The correct flowering time determines the reproductive success of higher plants. Extensive genetic studies in the last three decades using *Arabidopsis thaliana* have led to the discovery of four interdependent genetic pathways of flowering in plants: long‐day, autonomous, vernalization and gibberellin‐dependent (Boss *et al.,* 2004; Mouradov *et al.,* 2002; Simpson, 2002). Briefly, the long‐day and vernalization pathways are dependent on environmental factors, such as light and temperature, whereas the autonomous and gibberellin pathways seem to respond to endogenous signals. The flowering behaviour in Arabidopsis is determined by two genes, *FRIGIDA* (*FRI*) and *FLOWERING LOCUS C* (*FLC*), which negatively affect flowering (Choi *et al.,* 2011; Clarke and Dean, 1994; Gazzani *et al.,* 2003; Michaels *et al.,* 2004). More than one‐half of the flowering time variants in Arabidopsis accessions are accounted for by the allelic differences in FRI and FLC when the plants are grown under constant strongly flower‐promoting conditions without vernalizations (Guo *et al.,* 2012).

A plant’s response to pathogen infection shows some similarity to the response to abiotic stress. Both biotic and abiotic stresses involve cell death, ethylene, salicyclic acid (SA) and jasmonic acid (JA), and cause changes in the expression levels of some of the same transcription factors. One way in which plants respond to abiotic stresses is to accelerate their transition to reproduction (Korves and Bergelson, 2003). Changes in ethylene, SA and JA have been reported previously in potato spindle tuber viroid (PSTVd)‐infected tomato plants (Owens *et al.,* 2012). Often, viroid infection induces early flowering in plants, as reported previously in chrysanthemums infected with the chrysanthemum stunt viroid (CSVd) (Hosokawa *et al.,* 2004a). The infection of chrysanthemum by CSVd causes serious economic losses for 3 or 4 years after the initial infection by changing the flowering time, and also causes other symptoms, such as stunting and flower colour bleaching (Yoon *et al.,* 2014). Infected plants bloom earlier than normal plants of the same cultivar, and this effect increases with time (https://www.eppo.int/QUARANTINE/virus/Chrysanthemum_stunt/CSVD00_ds.pdf). Nevertheless, the key mechanistic question remains: ‘How does a highly structured, non‐coding RNA induce early flowering in its host?’

Viroids are not known to code for any protein. Hence, these infectious RNAs rely completely on host factors for their replication (Ding, 2009). On infection, viroids elicit the RNA silencing machinery as a result of either their highly base‐paired secondary structure or their double‐stranded RNA form that is generated during their replication. These viroid RNAs are processed by either the DICER or DICER‐LIKE (DCL) RNase III‐type ribonucleases, resulting in the production of small interfering RNAs (siRNAs) of 21–24 nucleotides, called viroid‐derived small RNAs (vd‐sRNAs) (Dadami *et al.,* 2013). The accumulation of such vd‐sRNAs is often observed in various viroid–host combinations (Bolduc *et al.,* 2010; Diermann *et al.,* 2010; Navarro *et al.,* 2009; Tsushima *et al.,* 2011). The profiling of such vd‐sRNAs reveals that the genomic (+) strands of the viroid produce more small RNA (sRNA) than do the antigenomic (–) strands. This can be attributed to the lower accumulation level of the (–) strands (Adkar‐Purushothama *et al.,* 2015a; Hutchins *et al.,* 1985; Wang *et al.,* 2011). This also suggests the possible involvement of both the (+) and (–) strand‐derived vd‐sRNAs in viroid pathogenicity. This hypothesis is supported by previous demonstrations using various host–viroid combinations (Adkar‐Purushothama *et al.,* 2015a, 2017b; Eamens *et al.,* 2014; Navarro *et al.,* 2012).

PSTVd and tomato (*Solanum lycopersicum *L. cv. Rutgers) plants present a good model for understanding the molecular basis of viroid–host relationships, given the fact that different PSTVd variants induce an array of symptoms in *S. lycopersicum *L. cv. Rutgers, ranging from mild to severe (Tsushima *et al.,* 2015). Hence, this viroid–host combination was chosen in order to understand the molecular basis of the early flowering observed during viroid infection. The results provide conclusive molecular and biological evidence that the silencing of *FRIGIDA‐like protein 3* (*FRL3*) by vd‐sRNAs induces early flowering in viroid‐infected plants.

## RESULTS

### PSTVd‐derived sRNAs are predicted to target the *FRL3* mRNA

PSTVd‐Mild (PSTVd‐M) induces mild symptoms in *S. lycopersicum* cv. Rutgers, whereas PSTVd‐Intermediate (PSTVd‐I) induces severe symptoms on infection (Tsushima *et al.,* 2015). Sequence analysis has shown that PSTVd‐M differs from PSTVd‐I by nine nucleotides: five substitutions, one deletion and three insertions (Tsushima *et al.,* 2011). The sRNAs recovered from plants infected with these PSTVd variants are of 21–24 nucleotides in length and are located throughout their genomes (Adkar‐Purushothama *et al.,* 2015a; Tsushima *et al.,* 2015). Previously, early flowering, relative to both PSTVd‐M‐ and mock‐infected plants, has been observed in PSTVd‐I‐infected plants. As PSTVd is a non‐coding RNA pathogen, the involvement of vd‐sRNA in this early flowering phenomenon can be envisaged.

**Figure 1:**
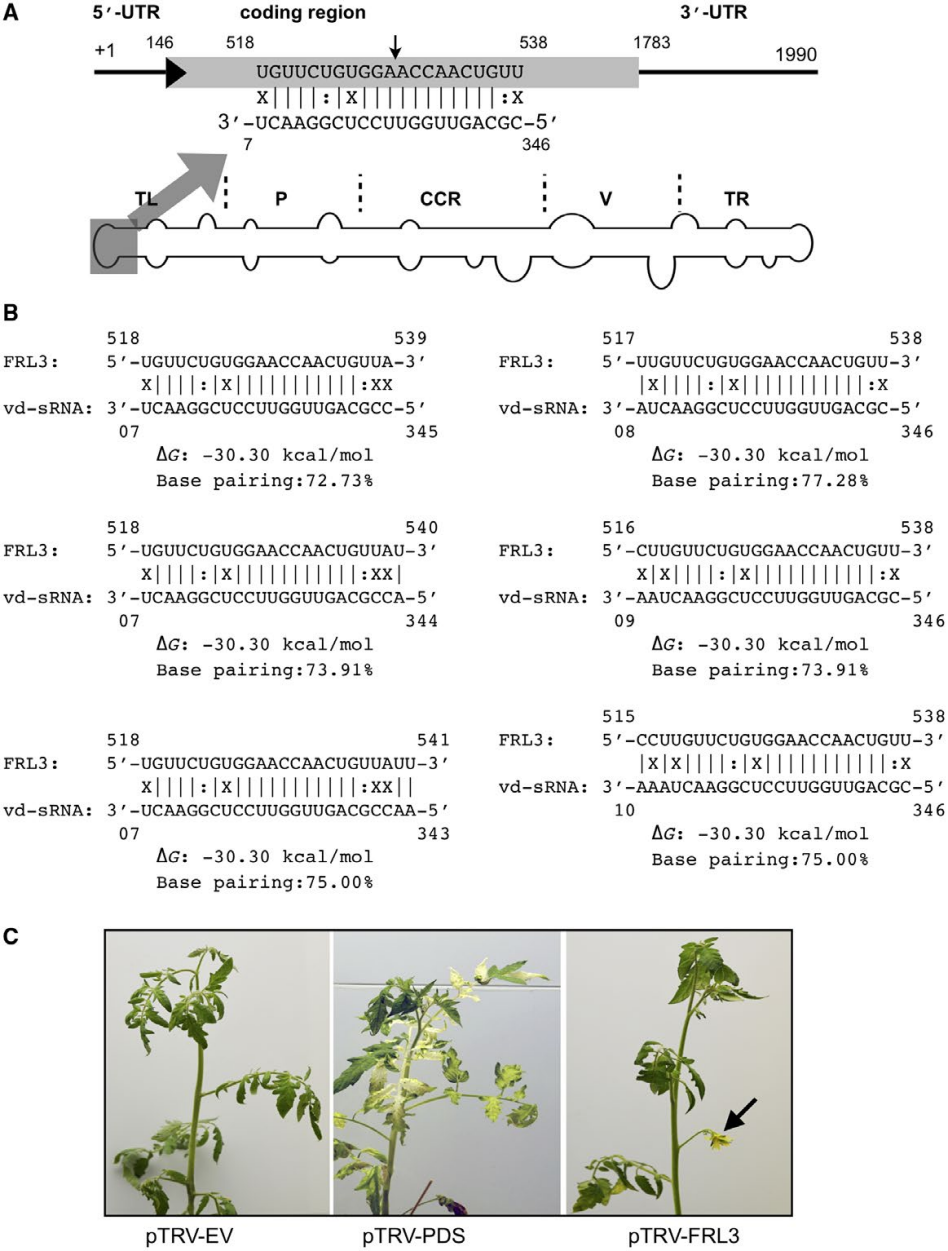
Schematic representation of the regions of potato spindle tuber viroid (PSTVd) predicted to target *FRIGIDA‐like protein 3* (*FRL3*) mRNA of tomato plants. (A) Schematic view of the PSTVd structure showing the five structural/functional domains namely, the terminal left (TL), pathogenicity (P), central conserved region (CCR), variable (V) and terminal right (TR). The viroid‐derived small RNA (vd‐sRNA) derived from the TL domain of the (+) strand of PSTVd was predicted to target the *FRL3* mRNA of tomato plants. The sequences are shown in the complementary polarity. UTR, untranslated region. (B) The duplexes predicted to form between the tomato *FRL3* mRNA targets and all of the possible vd‐sRNAs derived from PSTVd. (C) The tomato plants were subjected to a knockdown assay using a virus‐induced gene silencing (VIGS) technique in order to verify the roles of the *FRL3* mRNA in both the plant’s morphology and the induction of early flowering. At 30 days post‐infection (dpi), the plants exhibited phenotypic alterations. pTRV‐EV, plants agroinfiltrated with TRV2 empty vector; pTRV‐PDS, plants agroinfiltrated with pTRV:PDS; pTRV‐FRL3, plants agroinfiltrated with pTRV‐FRL3. [Colour figure can be viewed at wileyonlinelibrary.com]

Initially, both PSTVd variants were dissected *in silico* into 21‐nucleotide fragments, which were then used to interrogate publicly available tomato transcriptome datasets using the WMD3 web‐based tool (https://wmd3.weigelworld.org/cgi-bin/webapp.cgi). The resulting putative target sequences were analysed by BLAST, and the mRNAs which were related to flowering traits were selected for further analysis. Interestingly, vd‐sRNAs derived from the left terminal region (5′‐_346_CGCAGTTGGTTCCT_359‐1_CGGAACT_7_‐3′) of both PSTVd variants were predicted to target the open reading frame of the *FRL3 mRNA *(GenBank Acc. No. XM_010324803; Fig. 1A). *FRI* is required in order to increase the FLC transcript level, and this increase results in late flowering in Arabidopsis (Michaels and Amasino, 2001). The Gibbs minimal free energy (∆*G*), determined using PairFold, for the vd‐sRNA:FRL3 duplex is –30.30 kcal/mol (Andronescu, 2003). Figure 1B presents all of the possible 21–24‐nucleotide vd‐sRNAs that are predicted to form a duplex with the *FRL3 *mRNA of tomato. It is worth noting that all of these predicted 21–24‐nucleotide vd‐sRNA:FRL3 duplexes have the same ∆*G*, but differ in their base pairing percentages.

To evaluate the role of *FRL3* in the host’s phenotype, the *FLR3* gene of tomato was knocked down by virus‐induced gene silencing (VIGS). A portion of the *FRL3* gene amplified from tomato was ligated into the binary vector pTVR2 under the control of the 35S promoter. The binary vector containing the portion of the *FRL3* gene was named pTRV‐FRL3. pTRV‐PDS, which is capable of the down‐regulation of phytoene desaturase (PDS), was used as a positive control for the VIGS experiment, as the knockdown of PDS results in bleached leaves. Both of the binary vectors were then agroinfiltrated into tomato seedlings, as was the vector pTRV1 into the *Agrobacterium tumefaciens* strain GV3101, as described previously (Adkar‐Purushothama *et al.,* 2017a). Negative control plants were maintained by agroinfiltration with empty pTRV2 vector together with the vector pTRV1. At 21 days post‐infection (dpi), pTRV‐PDS plants exhibited slightly bleached leaves, which became more obvious at 28 dpi. The pTRV‐FRL3‐agroinfiltrated plants exhibited floral buds at 30 dpi, and fully expanded flowers at 35 dpi, whereas mock‐inoculated plants did not show any flower buds (Fig. 1C). These data reveal the involvement of the *FRL3* gene in early flowering in tomato plants.

### PSTVd‐I, but not PSTVd‐M, induces early flowering

**Figure 2:**
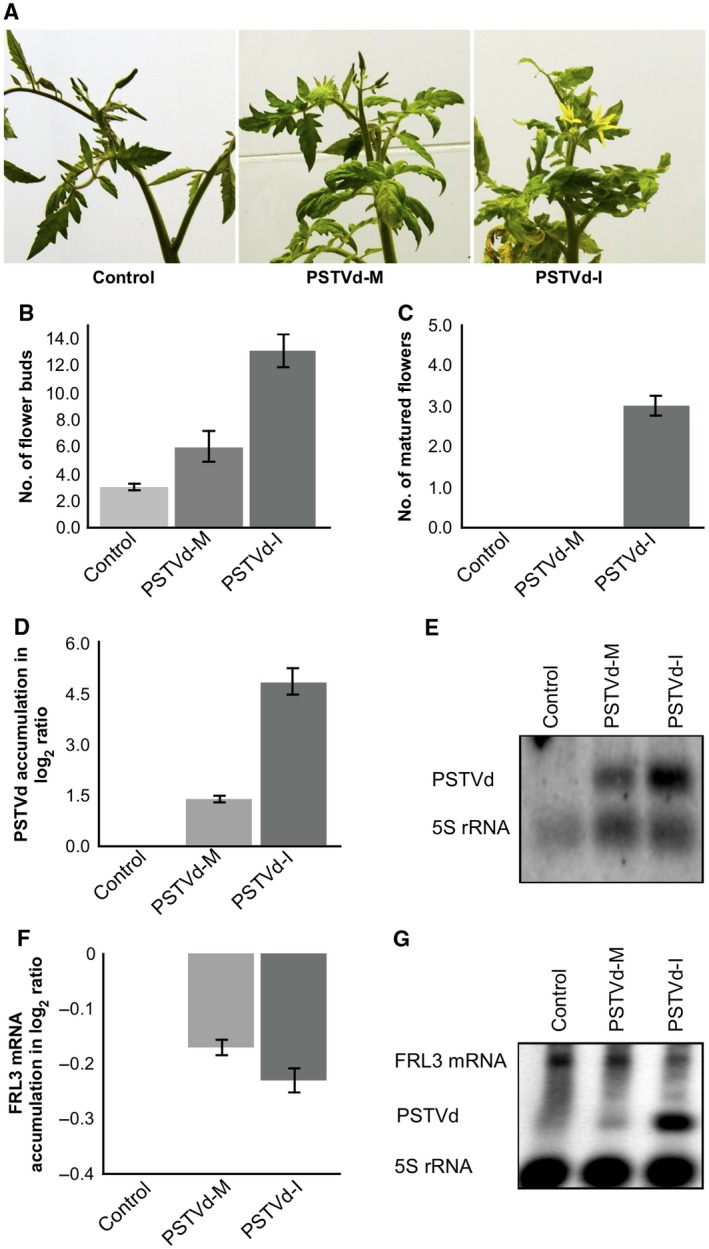
Potato spindle tuber viroid (PSTVd) infection induces early flowering in tomato plants. (A) The PSTVd variants PSTVd‐M (PSTVd‐Mild) and PSTVd‐I (PSTVd‐Intermediate) were inoculated into tomato plants cv. Rutgers. At 35 days post‐infection (dpi), the plants inoculated with PSTVd‐I exhibited mature flowers, whereas the PSTVd‐M‐inoculated plants showed flower buds similar to those of mock‐inoculated plants. The total number of flower buds (B) and fully opened flowers (C) observed in three separate experiments were recorded. The error bars indicate the standard deviation (SD). Total RNA extracted from tomato plants at 14 dpi was used to monitor the PSTVd titre by both reverse transcription–quantitative polymerase chain reaction (RT‐qPCR) (D) and RNA gel blot assay (E). The expression change is presented on a log_2_ scale. The error bars indicate the SD. The same RNA samples were used for the RT‐qPCR (F) and RNA gel blot assay (G) in order to monitor the effect of PSTVd variants on the expression level of *FRIGIDA‐like protein 3* (*FRL3*) mRNA in tomato plants. The expression change is presented on a log_2_ scale. The error bars indicate the SD. Each experiment was performed at least three times with true biological replicates. [Colour figure can be viewed at wileyonlinelibrary.com]

To understand the effect of PSTVd infection on the *FRL3* mRNA levels, tomato plants were inoculated with both PSTVd‐M and PSTVd‐I. The plants inoculated with PSTVd‐M exhibited initial symptoms at 14‐dpi, while those inoculated with PSTVd‐I showed symptoms, such as leaf curling and stunting, as early as 10‐dpi. At about 35‐dpi, the PSTVd‐M inoculated plants showed flower buds similar to those of mock‐inoculated plants, while the PSTVd‐I inoculated plants had fully opened flowers (Fig. 2A). Interestingly, viroid infected plants had a higher amount of flowers than mock‐inoculated plants. In other words, PSTVd‐M inoculated plants had a higher number of flower buds than those of the mock‐inoculated plants (Fig. 2B). Moreover, PSTVd‐I inoculated plants had a higher number of flowers than both the mock‐inoculated and the PSTVd‐M inoculated plants (Fig. 2C). At this point it is worth mentioning that FRL3 plays a role in early flowering while there may beother factors involved in determining the number of flowers in viroid infected plants. As low amounts of plants were used (n=9), further studies using a larger quantity of plants are required for statistical analysis to conclude the effect viroids have on the number of flowers.

To monitor both viroid accumulation and the *FRL3* mRNA level at the initial stages of infection, leaf samples were collected at 14 dpi. The viroid level at this stage was quantified by subjecting the total RNA extracted from these samples to reverse transcription‐quantitative polymerase chain reaction (RT‐qPCR) using PSTVd‐specific primers (Boonham *et al.,* 2004). As shown in Fig. 2D, PSTVd‐I accumulated to a greater extent than PSTVd‐M. The differential accumulation of PSTVd‐M and PSTVd‐I was further validated by RNA gel blot assays using a PSTVd‐specific probe (Fig. 2E).

In order to determine the effect of these PSTVd variants on the expression level of *FRL3* mRNA, RT‐qPCR analysis was performed on the RNA samples used to quantify viroid accumulation employing *FRL3* gene‐specific primers. The expression levels observed in mock‐inoculated plants were used for normalization. The plants inoculated with PSTVd‐I exhibited a higher degree of down‐regulation of *FRL3* mRNA relative to PSTVd‐M‐inoculated plants (Fig. 2F). This difference in the amount of *FRL3* mRNA down‐regulation may be a result of the higher accumulation of PSTVd‐I relative to PSTVd‐M. The data clearly show the repression of *FRL3* mRNA in the viroid‐infected plants, regardless of the variant used. In addition, the repression of *FRL3* observed by RT‐qPCR was further validated by RNA gel blot assay using *FRL3*‐specific probes (Fig. 2G). These data are in agreement with the RT‐qPCR results, and thus further support the hypothesis that PSTVd infection down‐regulates the accumulation of *FRL3* mRNA in tomato plants.

### Accumulation of sRNA targeting *FRL3 *mRNA and cleavage of *FRL3* mRNA in infected plants

**Figure 3:**
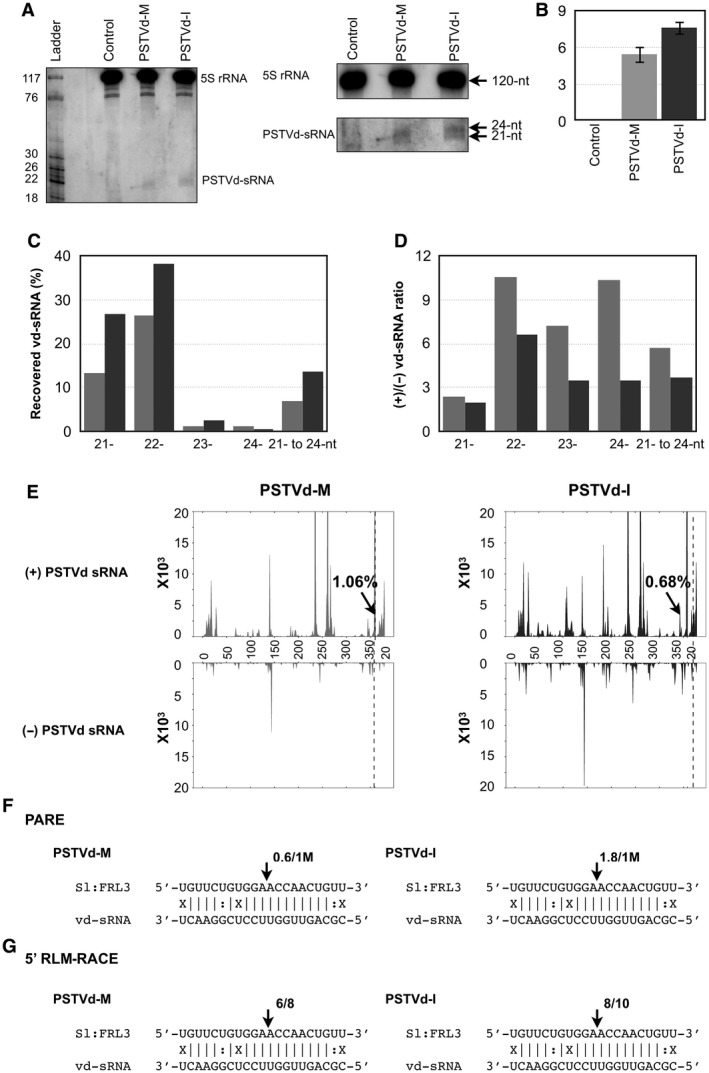
Sequence profile of the potato spindle tuber viroid small RNAs (PSTVd‐sRNAs) recovered from infected tomato plants. (A) Total RNAs extracted from tomato plants at 21 days post‐infection (dpi) were used to monitor the PSTVd‐sRNA levels by RNA gel blot assay employing radiolabelled PSTVd probes. The 5S rRNA was used as a loading control. Left panel: enlarged view of gel blot assay. (B) The gel blot signals quantified and expressed as a ratio of the viroid‐derived small RNA (vd‐sRNA) to the 5S rRNA signals. For each set of experiments, the ratio of target mRNA to 5S rRNA obtained with mock‐inoculated plants were set at a value of zero. The additional bars indicate the relative vd‐sRNA/5S rRNA ratio for each PSTVd variant (as indicated) expressed. Each experiment was performed at least three times. Error bars indicate standard deviation (SD). (C) Size distribution of sRNAs derived from the PSTVd variants. The horizontal axis indicates the lengths of the sRNAs, and the vertical axis indicates the total percentage of the sRNA reads. The grey bars are PSTVd‐M (PSTVd‐Mild) and the black bars are PSTVd‐I (PSTVd‐Intermediate). nt, nucleotide. (D) Comparison of the ratios of (+) and (–) strand‐derived sRNAs recovered from PSTVd‐M‐ and PSTVd‐I‐inoculated plants. The horizontal axis indicates the lengths of the sRNAs, and the vertical axis indicates the ratios of sRNA reads. The grey bars are PSTVd‐M and the black bars are PSTVd‐I. (E) Profiles of the PSTVd‐sRNA populations recovered from tomato plants infected with PSTVd‐M and PSTVd‐I. The top panel shows the profile of the PSTVd‐sRNAs derived from the (+) strand, and the bottom panel presents those from the (–) strand. The regions of vd‐sRNA targeting the *FRIGIDA‐like protein 3* (*FRL3*) gene are indicated with an arrow. The first 20 nucleotides of PSTVd are repeated in order to show vd‐sRNA targeting the *FRL3* gene. The monomeric length of PSTVd RNA is separated by a vertical dotted line. (F, G) *FRL3* mRNA/vd‐sRNA duplexes predicted to be formed by the sRNAs derived from the PSTVd‐M and PSTVd‐I variants. The arrows indicate the 5′ termini of the *FRL3* mRNA fragments isolated from the PSTVd‐infected plants, as identified by parallel analysis of RNA ends (PARE) (F) and 5′ RNA ligase‐mediated rapid amplification of cDNA ends (5′ RLM‐RACE) (G) products, with the frequency of the clones being shown (e.g. 0.6/1M, indicates that 0.6 cleavage products were found in 1 million reads, whereas 6/8 indicates that six cleavage products were found in eight analysed clones). The sequences are shown in complementary polarity.

To verify the production of sRNA by the PSTVd variants in tomato plants, and to investigate the accumulation levels of *FRL3* mRNA targeting vd‐sRNAs, total RNA extracted from both leaf and stem samples at 21 dpi was subjected to high‐throughput sequencing. Approximately 11–12 million reads of sRNA, ranging from 21 to 24 nucleotides in length, were obtained from the leaf and stem samples of mock‐, PSTVd‐M‐ and PSTVd‐I‐inoculated plants (Table 1). Detailed analysis with the sRNA recovered from both PSTVd‐M‐ and PSTVd‐I‐inoculated plants revealed higher numbers of vd‐sRNAs in PSTVd‐I‐ than in PSTVd‐M‐inoculated plants. More specifically, the raw data revealed 620 651 reads (6.84%) of vd‐sRNA in PSTVd‐M‐infected plants compared with 1 154 772 reads (13.52%) of vd‐sRNA in PSTVd‐I‐infected plants. In order to verify the differential recovery of vd‐sRNA in PSTVd‐M‐ and PSTVd‐I‐inoculated plants, an RNA gel blot assay was performed to detected vd‐sRNA accumulation. As presented in Fig. 3A,B, there was a greater amount of vd‐sRNA in PSTVd‐I‐ than in PSTVd‐M‐inoculated plants. This result is in agreement with the vd‐sRNA recovered by deep sequencing. The lower recovery of vd‐sRNA from PSTVd‐M‐inoculated plants is presumably a result of the lower accumulation level of this viroid variant relative to that of the PSTVd‐I variant.

**Table 1:** Summary of small RNAs (sRNAs) identified by high‐throughput sequencing.

Samples	Total sRNA	PSTVd‐sRNA	% vd‐sRNA	(+) sRNA	(–) sRNA	+/– ratio
Healthy	7 856 247	229/232*				
PSTVd‐M	9 078 067	620 651	6.84	528 670	91 981	5.75
PSTVd‐I	8 541 046	1 154 772	13.52	906 597	248 175	3.65

vd‐sRNA, viroid‐derived small RNA.

^*^229 and 232 sRNAs of the potato spindle tuber viroid‐Mild (PSTVd‐M) and PSTVd‐Intermediate (PSTVd‐I) types, respectively, that were 100% identical to the (+) and (–) strands of PSTVd‐M and PSTVd‐I.

The recovered vd‐sRNAs from both PSTVd‐M‐ and PSTVd‐I‐inoculated plants were analysed for both their size and distribution on the viroid genomes. As shown in Fig. 3C, PSTVd‐I‐inoculated plants produced more vd‐sRNAs than did PSTVd‐M‐inoculated plants for the 21‐, 22‐ and 23‐nucleotide sRNAs. In both viroid infections, greater amounts of vd‐sRNAs corresponding to the (+) strand were recovered. However, plants inoculated with PSTVd‐M showed a higher (+)/(–) ratio than did those inoculated with PSTVd‐I (Fig. 3D). All of the recovered vd‐sRNAs were profiled on both the (+) and (–) strands of the respective PSTVd variants in order to understand the production of the vd‐sRNAs in the TL domain of both viroids (Fig. 3E). Analysis of the total number of vd‐sRNAs targeting the *FRL3* gene revealed that PSTVd‐M produced 1.06% (5610 vd‐sRNAs of 528 670 recovered (+) sRNAs) PSTVd‐sRNAs, whereas PSTVd‐I produced 0.68% (6159 vd‐sRNAs of 906 597 recovered (+) sRNAs) vd‐sRNAs (Table 2). The data presented here demonstrate that infection with both PSTVd variants does indeed result in the production of the vd‐sRNAs predicted above, and that these vd‐sRNAs can target the *FRL3* mRNA.

**Table 2:** Summary of small RNAs (sRNAs) identified by high‐throughput sequencing that are predicted to bind the *FRIGIDA‐like protein 3* (*FRL3*) mRNA of tomato plants.

vd‐sRNA	Position	vd‐sRNA sequence	PSTVd‐M (%)*	PSTVd‐I (%)*
21	346‐07	CGCAGTTGGTTCCTCGGAACT	1.86	0.87
22	345‐07	CCGCAGTTGGTTCCTCGGAACT	0.25	0.20
22	346‐08	CGCAGTTGGTTCCTCGGAACTA	0.43	0.24
23	344‐07	ACCGCAGTTGGTTCCTCGGAACT	3.05	3.03
23	346‐09	CGCAGTTGGTTCCTCGGAACTAA	0.79	0.65
24	343‐07	AACCGCAGTTGGTTCCTCGGAACT	0.38	0.55
24	346‐10	CGCAGTTGGTTCCTCGGAACTAAA	0.22	0.20
**Total**			**1.06**	**0.68**

PSTVd‐M, potato spindle tuber viroid‐Mild; PSTVd‐I, potato spindle tuber viroid‐Intermediate; vd‐sRNA, viroid‐derived small RNA.

^*^(+) sRNA was used for the calculation.

The direct cleavage of a target RNA by a microRNA (miRNA) is often confirmed using 5′ RNA ligase‐mediated rapid amplification of cDNA ends (5′ RLM‐RACE) (Thomson *et al.,* 2011). However, recently, 5′ RLM‐RACE coupled with high‐throughput sequencing, such as the parallel analysis of RNA ends (PARE), has gained popularity as this technique allows the evaluation of a large number of samples in a single experiment (German *et al.,* 2009). Moreover, the latter method demonstrates the degree of target mRNA cleavage under different conditions (Adkar‐Purushothama *et al.,* 2017b). To verify the extent of *FRL3* mRNA cleavage that occurred in both PSTVd‐M‐ and PSTVd‐I‐infected plants, the total RNA extracted at 21 dpi was high‐throughput sequenced after the addition of an adapter to the 5′ uncapped region, as described previously (Adkar‐Purushothama *et al.,* 2017b). Both the PSTVd‐M‐ and PSTVd‐I‐inoculated plants revealed cleavage in the predicted target region (Fig. 3F).

To add physical support to this conclusion, a 5′ RLM‐RACE experiment was performed on the 21‐dpi RNA preparations using an *FRL3* mRNA gene‐specific nested PCR experiment (Adkar‐Purushothama *et al.,* 2015a). Almost all of the *FRL3* transcripts had 5′ termini identical to that of the predicted cleavage site, more specifically located between positions 10 and 11 from the 5′ termini (Fig. 3G). No PCR amplification was obtained when similar experiments were performed with RNA preparations obtained from mock‐inoculated plants (data not shown), confirming the specific cleavage of the *FRL3* mRNA by PSTVd‐sRNA. The data presented here validate the cleavage of the predicted *FRL3* mRNA sequence in PSTVd‐infected plants. Taken together, these data confirm that both PSTVd variants are capable of producing vd‐sRNAs that are predicted to form a duplex with the *FRL3* mRNA, as well as the cleavage of the predicted target mRNAs in the plants.

### Transient expression of vd‐sRNA induces early flowering in tomato plants

**Figure 4:**
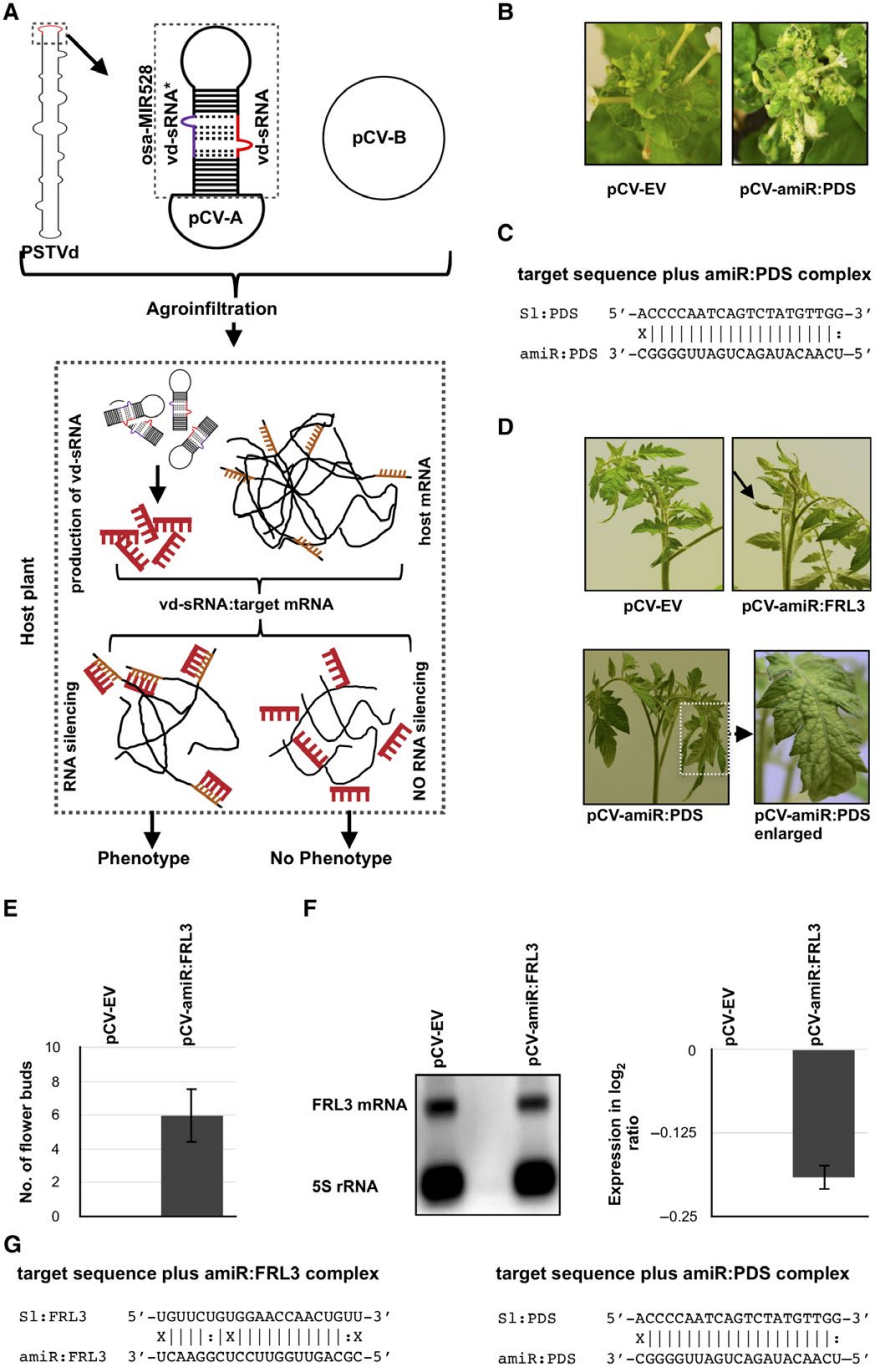
Transient expression of viroid‐derived small RNA (vd‐sRNA) induces phenotypic changes in tomato cv. Rutgers. (A) Flow chart illustrating the details of the vd‐sRNA:virus‐induced gene silencing (VIGS) experiment. The vd‐sRNA expressed as an artificial microRNA (amiRNA) in the pCV‐A vector was agroinfiltrated, together with pCV‐B, into plants at the cotyledon stage. The specific interaction of vd‐sRNA with the target sequence leads to either RNA‐induced silencing complex (RISC)‐mediated cleavage or translational repression, which, in turn, results in the phenotype of the plant. (B) The vd‐sRNA:VIGS assay was verified by agroinfiltration of pCV‐amiR:PDS into *Nicotiana benthamiana* plants. The resulting plants showed bleaching at 35 days post‐infection (dpi). (C) Duplexes predicted to be formed by the complexes of vd‐sRNAs and their target sequences. (D) The tomato plants were subjected to a vd‐sRNA:VIGS assay in order to evaluate the ability of the vd‐sRNA to induce early flowering. At 35 dpi, plants agroinfiltrated with pCV‐amiR:FRL3 exhibited early flower buds. pCV‐EV, plants agroinfiltrated with pCV emply vector; pCV‐amiR:PDS, plants agroinfiltrated with pCV‐amiR:PDS, which is capable of inducing the RNA silencing of the phytoene desaturase (PDS) mRNA; pCV‐amiR:FRL3, plants agroinfiltrated with pCV‐amiR:FRL3, which is capable of producing sRNA similar to the vd‐sRNA that was predicted to bind to *FRIGIDA‐like protein 3* (*FRL3*) mRNA. (E) The total number of flowers observed in three separate experiments were recorded. The error bars indicate the standard deviation (SD). (F) Total RNA extracted from systemic leaf samples from the agroinfiltrated plants was subjected to an RNA gel blot assay in order to evaluate the effect of vd‐sRNA on the *FRL3* mRNA using *FRL3*‐specific radiolabelled probes. The resulting gel blot signals (from the right panel) were quantified and expressed as a log_2_ ratio of *FRL3* mRNA to the 5S rRNA signal. (G) Duplexes predicted to be formed by complexes of vd‐sRNA and their target sequences. [Colour figure can be viewed at wileyonlinelibrary.com]

In order to demonstrate the direct role of the putative PSTVd‐sRNAs on early flowering in tomato plants, a vd‐sRNA delivery system was developed by modifying the miRNA‐mediated virus‐induced gene silencing (MIR VIGS) strategy developed previously to study functional genomics in *Nicotiana benthamiana *(Tang *et al.,* 2010). In MIR VIGS, a modified cabbage leaf‐curl geminivirus vector was used to express artificial and endogenous miRNAs on the miR319 backbone in the plant system in order to silence the expression of endogenous genes. Here, an artificial microRNA (amiRNA) is constructed using the sequenced vd‐sRNA, and is then ligated into the binary vector pCV‐A under the control of the 35S promoter. On agroinfiltration together with the vector pCV‐B, the former vector is capable of producing vd‐sRNAs similar to those produced by the viroid, and these, in turn, bind to their target mRNA sequences. If the vd‐sRNA produced by amiRNA binds the target, it induces morphological changes in the host plant, as binding leads to either RNA‐induced silencing complex (RISC)‐mediated cleavage or translational repression (Fig. 4A). In order to evaluate the effect of MIR VIGS with a MIR528 backbone, the amiRNA was constructed using an osa‐MIR528 sequence as backbone, as performed previously (Adkar‐Purushothama, *et al.,* 2017b). The 21‐nucleotide sequence of *PDS* mRNA was synthesized by PCR and ligated into the binary vector pCV‐A under the control of the 35S promoter (pCV‐amiR:PDS). On agroinfiltration together with the pCV‐B vector, pCV‐amiR:PDS is capable of producing sRNA that can bind to its complementary sequence of PDS mRNA and trigger RNA silencing of the PDS gene, which, in turn, induces a bleaching effect (Adkar‐Purushothama and Perreault, 2018; Tang *et al.,* 2010). At approximately 35 dpi, agroinfiltrated *N. benthamiana* plants exhibited bleaching of their leaves (Fig. 4B). In order to rule out an effect of the vectors on the morphology of the plants, a control experiment was performed by co‐expression of the empty pCV‐A vector with pCV‐B. None of the plants agroinfiltrated with empty vector showed any phenotypic changes. The amiR:PDS/PDS target complex predicted to be formed by the interaction between amiRNA and the putative PDS mRNA target sequences is shown in Fig. 4C. These data indicate the feasibility of the vd‐sRNA:VIGS technique in *N. benthamiana* plants using the osa‐MIR528 sequence as backbone.

In order to demonstrate the role of putative vd‐sRNAs on both *FRL3* mRNA and the host’s phenotype, an amiRNA sequence for vd‐sRNA targeting *FRL3* was constructed by PCR on the osa‐MIR528 backbone. The resulting amiR‐FRL3 was ligated into the binary vector pCV‐A under the control of the 35S promoter, as described previously (pCV‐amiR‐FRL3). On agroinfiltration, this vector is capable of producing vd‐sRNAs similar to those produced by the viroid, and these, in turn, bind to the *FRL3* mRNA. Positive control plants were maintained by agroinfiltration with pCV‐amiR‐PDS, whereas negative control plants were agroinfiltrated with both empty pCV‐A vector and pCV‐B vector. At approximately 35 dpi, plants agroinfiltrated with pCV‐amiR‐PDS showed mild photobleaching, whereas plants agroinfiltrated with pCV‐amiR:FRL3 exhibited flower buds but mock‐inoculated plants did not. None of the positive or negative controls showed early flowering (Fig. 4D,E). RNA gel blot assays performed on the total RNA extracted from pCV‐amiR:FRL3 revealed a slight repression of the *FRL3* mRNA level relative to that of the control (pCV‐EV) plants (Fig. 4F). Both the amiR:FRL3/*FRL3* target and the amiR:PDS/PDS target complexes predicted to be formed by the interaction between amiRNA and the putative PDS mRNA target sequences are shown in Fig. 4G. These data confirm the direct effect of vd‐sRNA in both the down‐regulation of the *FRL3* transcripts of tomato plants and the induction of early flowering.

## DISCUSSION

Pathogens, such as bacteria, oomycetes and viruses, induce early flowering in infected plants (Korves, 2003; Yamagishi *et al.,* 2016). For example, infection by *Pseudomonas syringae* and *Xanthomonas campestris* induces early flowering in infected Arabidopsis (Yamagishi *et al.,* 2016). Similarly, CSVd is known to induce early flowering in susceptible chrysanthemum cultivar (Hosokawa *et al.,* 2004b; Yoon *et al.,* 2014). As viroids are a non‐coding RNA pathogen, the role of vd‐sRNA in the early flowering of chrysanthemum plants was suspected. However, because of the unavailability of chrysanthemum genome data at this time and the observation of early flowering in PSTVd‐infected tomato plants, it was decided to use the PSTVd–tomato system instead of CSVd–chrysanthemum. As viroids are known to produce sRNAs throughout both their (+) and (–) strands, and these vd‐sRNAs are known to induce RNA silencing of host transcripts, all of the 21‐nucleotide‐long sRNAs derived from both PSTVd‐M and PSTVd‐I were interrogated against the tomato genome in order to determine whether there was any complementarity between the PSTVd‐sRNA and the flowering/reproductive‐related genes of tomato plants. One of the PSTVd‐sRNAs derived from both variants (sRNAs derived from the nucleotide positions 348 to 7 and 346 to 7 of PSTVd‐M and PSTVd‐I, respectively) was predicted to interact with a section of the open reading frame of the *FRL3* mRNA (Fig. 1A). Previously, Choi et al. (2005) isolated an early‐flowering *Arabidopsis* mutant, *suppressor of FRIGIDA3 *(*suf3*). The *suf3* mutation caused a decrease in the transcript level of *FLC*, thus inducing early flowering. Although *suf3* revealed only a partial reduction of the *FLC *transcript level, it largely suppressed the late‐flowering phenotype (Choi *et al.,* 2005). In other words, the production of FRI induces FLC production, which, in turn, delays flowering (Michaels and Amasino, 2001). The knockdown of *FRL3* mRNA in tomato plants by VIGS induced early flowering in these plants, indicating the involvement of *FRL3* mRNA in the flowering time in tomato plants (Fig. 1B).

In order to verify the effect of PSTVd infection on early flowering in tomato, plants were inoculated with transcripts of both PSTVd‐M and PSTVd‐I (Fig. 2). The PSTVd‐I‐inoculated plants exhibited fully opened flowers at 35 dpi, and the PSTVd‐M‐inoculated plants showed a greater amount of flower buds than those seen in control plants at this point. However, it should be noted that tomato plants are annuals and early flowering is not as obvious as in chrysanthemum plants. This is mainly because chrysanthemum plants are perennials and, after 3–4 years of CSVd infection, they flower at least 2 weeks earlier than do healthy plants (Yoon *et al.,* 2014). As presented in Fig. S1 (see Supporting Information), PSTVd‐M‐infected tomato plants exhibited flowering times similar to those of mock‐inoculated plants, whereas PSTVd‐I‐infected plants showed mature flowers at the same time (35 dpi). PSTVd‐RG1 variants are known to accumulate more rapidly than either PSTVd‐M or PSTVd‐I variants, and plants infected with this variant show severe disease symptoms with stunted growth and an increased number of lateral branches (Adkar‐Purushothama *et al.,* 2017a,2017b). However, PSTVd‐RG1‐inoculated plants did not show any flower buds, although PSTVd‐RG1 is capable of producing vd‐sRNA against *FRL3* mRNA, similar to PSTVd‐M and PSTVd‐I variants. The accumulation of PSTVd and the expression levels of the *FRL3* transcripts were monitored by RT‐qPCR using total RNA extracted at 14 dpi. In PSTVd‐I‐inoculated plants, a greater accumulation of viroid was detected relative to that in PSTVd‐M‐inoculated plants, in agreement with previous results (Adkar‐Purushothama *et al.,* 2015a). In addition, PSTVd‐I‐inoculated plants showed early flower bud formation, a greater number of flowers and stronger down‐regulation of *FRL3* transcripts compared with PSTVd‐M‐ and mock‐inoculated plants. Taken together, these data show that a higher accumulation of viroid has a greater negative effect on *FRL3* transcripts, which, in turn, results in early flowering.

As the direct involvement of vd‐sRNA in the induction of the negative effect on *FRL3* transcripts was suspected, it was important to verify the production of vd‐sRNA in the viroid‐inoculated plants. Hence, sRNAs obtained from both shoot and leaf samples of PSTVd‐M‐ and PSTVd‐I‐inoculated plants at 21 dpi were sequenced (Fig. 3A). The PSTVd‐I‐inoculated plants produced greater amounts of vd‐sRNA than found in PSTVd‐M‐inoculated plants. This may be a result of the greater accumulation level of PSTVd‐I. Mapping of the vd‐sRNAs on the respective viroid (+) and (–) strands showed similar profiles (Fig. 3D). These results are in agreement with previous vd‐sRNA profiles using the same viroid variant–host system (Adkar‐Purushothama *et al.,* 2015a; Tsushima *et al.,* 2015). However, these data also showed a direct relationship between the PSTVd accumulation level, the amount of *FRL3*‐binding vd‐sRNAs and the negative effect induced by the PSTVd variant on the *FRL3* transcript in tomato plants. PARE was performed with the aim of validating the RNA interference (RNAi)‐induced cleavage of the *FRL3* transcript in viroid‐infected plants (Fig. 3E). Although cleavage at the predicted cleavage sites of the vd‐sRNA:FRL3 duplex was found, the high degree of cleavage obtained for an miRNA:target duplex was not observed (German *et al.,* 2009). Hence, 5’ RLM RACE was performed in order to verify the PARE data. The data revealed the cleavage of the *FRL3* transcript at the predicted cleavage site (Fig. 3F). These results further support the hypothesis that vd‐sRNA induces RNAi‐mediated cleavage of the *FRL3* transcript.

To demonstrate the direct role of PSTVd‐sRNAs on early flowering in tomato plants, a vd‐sRNA delivery system was developed by modifying the MIR VIGS strategy previously developed to study functional genomics in *N. benthamiana* (Tang *et al.,* 2010). This system was named vd‐sRNA:VIGS, as this technique transiently expresses vd‐sRNA (Fig. 4A). The original method used the construction of amiRNA on the miR319 backbone. Here, the miR319 backbone was replaced with the osa‐MIR528 backbone because: (i) the construction of amiRNA on miR319 requires multiple PCRs; and (ii) the transient expression of amiRNA on an osa‐MIR528 backbone in tomato plants has been developed previously in order to deliver vd‐sRNA (Adkar‐Purushothama *et al.,* 2015a, 2017b). The transient expression of amiRNA on the osa‐MIR528 backbone was evaluated in both *N. benthamiana* and tomato plants by expressing amiRNA directed against PDS. The PSTVd‐sRNA that was predicted to form a duplex with the *FRL3* transcript of tomato was ligated into the MIR VIGS vector and then agroinfiltrated into tomato plants (Fig. 4). At approximately 35 dpi, pCV‐amiR‐FRL3 vector‐agroinfiltrated tomato plants exhibited flower buds, whereas control plants did not. An RNA gel blot assay reveled a decrease in *FRL3* mRNA levels in pCV‐amiR‐FRL3‐agroinfiltrated plants. These data support the concept of a direct effect of the predicted vd‐sRNA on the *FRL3* mRNA level in tomato plants, and that vd‐sRNAs are involved in early flowering. Previously, amiRNA capable of expressing vd‐sRNA in transgenic *N. benthamiana* was developed in order to show the direct effect of vd‐sRNA on the phenotype of the host (Eamens *et al.,* 2014). However, the production of transgenic plants is laborious and time consuming. Hence, the vd‐sRNA:VIGS technique developed for the transient expression of vd‐sRNAs will be very useful in the study of the role of vd‐sRNAs in the phenotype of the host.

In summary, PSTVd was used as a model viroid with which to answer the question: ‘How do viroids induce early flowering in susceptible hosts such as tomato plants?’ The vd‐sRNA:VIGS technique demonstrated in this study will help researchers to evaluate the role of individual vd‐sRNAs in the phenotype of the host.

## EXPERIMENTAL DETAILS

### PSTVd transcripts and viroid bioassay

The dimeric constructs of PSTVd‐M (GenBank Acc. No. AB623143) and PSTVd‐I (GenBank Acc. No. AY937179) were used to synthesize infectious dimeric transcripts as described previously (Adkar‐Purushothama *et al.,* 2015a). PSTVd RNA transcripts (250 ng) of the respective viroid variants were inoculated into tomato plants (*Solanum lycopersicum* cv. Rutgers; Livingston Seed Co., Columbus, OH, USA), which were maintained in a growth chamber as described previously (Adkar‐Purushothama *et al.,* 2015a).

### RNA purification and RT‐qPCR

Total RNA from leaf samples was extracted, and cDNA was synthesized, as described previously (Adkar‐Purushothama *et al.,* 2015a). For the evaluation of both the PSTVd titre and the gene expression levels of *FRL3*, 10 ng of this cDNA was used in qPCR experiments together with the appropriate primer combinations (Table S1, see Supporting Information). The RT‐qPCR results were normalized to three housekeeping genes: the ARF‐like GTPase family protein (ASAR1), the transducing/WD40 repeat family protein and the ubiquitin‐conjugating enzyme (UBC). A negative control was maintained for every primer pair, and these were consistently negative. The relative expression levels were calculated using the qBASE framework (Hellemans *et al.,* 2007). All of the RT‐qPCR analyses were performed commercially at the Laboratoire de Génomique Fonctionnelle de l’Université de Sherbrooke, Sherbrooke, QC, Canada (https://palace.lgfus.ca).

### Small RNA extraction, high‐throughput sequencing and data analysis

For the high‐throughput sequencing of sRNAs, sRNAs of 15–50 nucleotides in length were purified from total RNA extracted from 21‐dpi plants and subjected to sRNA library preparation commercially at Hokkaido System Science Co. (Hokkaido, Japan) using a Genetic Analyzer IIx (Illumina, San Diego, California, USA). All of the samples were processed simultaneously in the Illumina system using an index sequence. Adapter sequences were removed from the resulting raw data, and sequences of 21–24 nucleotides were grouped by mapping onto the (+) and (–) strands of the respective PSTVd variants (Adkar‐Purushothama, *et al.,* 2015b). The resulting reads were then plotted on either the (+) or (–) strand of both PSTVd‐M and PSTVd‐I using the standard pattern matching algorithm after normalizing to the reads per million (RPM) scale (Adkar‐Purushothama *et al.,* 2017a).

### PARE library construction, data analysis and 5′ RLM‐RACE

For the PARE analysis, the PARE library previously constructed on total RNA obtained from 21‐dpi plants was used (Adkar‐Purushothama *et al.,* 2017a). The adapter sequences were trimmed from the raw data, and only 20‐nucleotide‐long sequences were filtered. The filtered sequences were used to search for the cleavage site of the *FRL3* transcript.

For 5’ RLM‐RACE, a previously described RNA adapter was ligated to 10 μg of total RNA, as described previously (Adkar‐Purushothama *et al.,* 2015a; Navarro *et al.,* 2012). The resulting product was reverse transcribed using an *FRL3*‐specific primer, followed by nested PCR (Table S1). The nested PCR products were separated by 2.0% agarose gel electrophoresis, and the gel fragment containing the band corresponding to the expected amplicon was cut out and the RNA was eluted using SpinX columns (Corning Incorporated, Corning, New York, USA). The purified products were cloned into the pGEM‐T easy vector (Promega , Madison, WI, USA), and commercially sequenced. The resulting sequences were analysed using the CLC Free Workbench version 4.6 software (https://www.clcbio.com/index.php?id=28).

### Construction of VIGS and vd‐sRNA:VIGS vectors and agroinfiltration

To generate the pTRV2 derivative targeting *FRL3*, the cDNA of *FRL3* mRNA was amplified from tomato cv. Rutgers using RT‐PCR with gene‐specific primers (Table S1). The resulting amplicons were cloned into the pGEM‐T easy vector (Promega), and this cloning was confirmed by sequence analysis. The product was then subcloned into the binary vector pTRV2. The resulting binary vector was then transformed into the *A. tumefaciens* strain GV3101, and this was then used for agroinfiltration, as described previously (Adkar‐Purushothama *et al.,* 2017a).

To evaluate the role of vd‐sRNA on both the *FRL3 *mRNA levels and the phenotype of the tomato plant (induction of early flowering), amiRNAs with vd‐sRNA sequences were synthesized on an osa‐MIR528 backbone by PCR (Table S1). The resulting amplicons were cloned into the pGEM‐T easy vector (Promega), and this cloning was confirmed by sequence analysis. The product was then subcloned into the binary vector pCV‐A after digesting both of the plasmids with the restriction endonucleases *Xba*I and *Kpn*I. The resulting binary vectors were then transformed into an *A. tumefaciens* strain GV3101, and this was then used for agroinfiltration into tomato plants. Agroinfiltrated plants were grown at 23 ºC with 16 h of light and 8 h of darkness.

### Probe preparation and RNA gel blot assay

In order to detect the expression of both *FRL3* mRNA and 5S rRNA, riboprobes were prepared using the genes previously amplified from tomato cv. Rutgers and cloned into the pBluesScript KS(+) vector. The details of the primers used to amplify the genes are presented in Table S1. The dimeric PSTVd‐I construct was used to produce the (–) PSTVd riboprobe, which was used to detect PSTVd. In order to prepare the riboprobes, either the T3 or T7 MAXIscript kit (Ambion, Carlsbad, California, USA) was used after linearizing the plasmid with appropriate restriction endonucleases. In order to detect both the host’s genes and the vd‐sRNA, 5.0 and 10 µg of the total RNA samples were used. For the detection of vd‐sRNA, total RNA was separated by 12% denaturation polyacrylamide gel electrophoresis. The RNA gel blots were performed as described previously (Adkar‐Purushothama *et al.,* 2015a).

## Supporting information


**Fig S1** Effect of potato spindle tuber viroid (PSTVd) variants in tomato plants at 35 days post‐infection (dpi).

**Table S1** Primers used in this study.
