# Supplementary figures and images for: Viroid‐derived small RNA induces early flowering in tomato plants by RNA silencing
Source: Mol Plant Pathol. 2018 Sep 28;19(11):2446–58. doi: 10.1111/mpp.12721 (PMC6637976; doi:10.1111/mpp.12721)

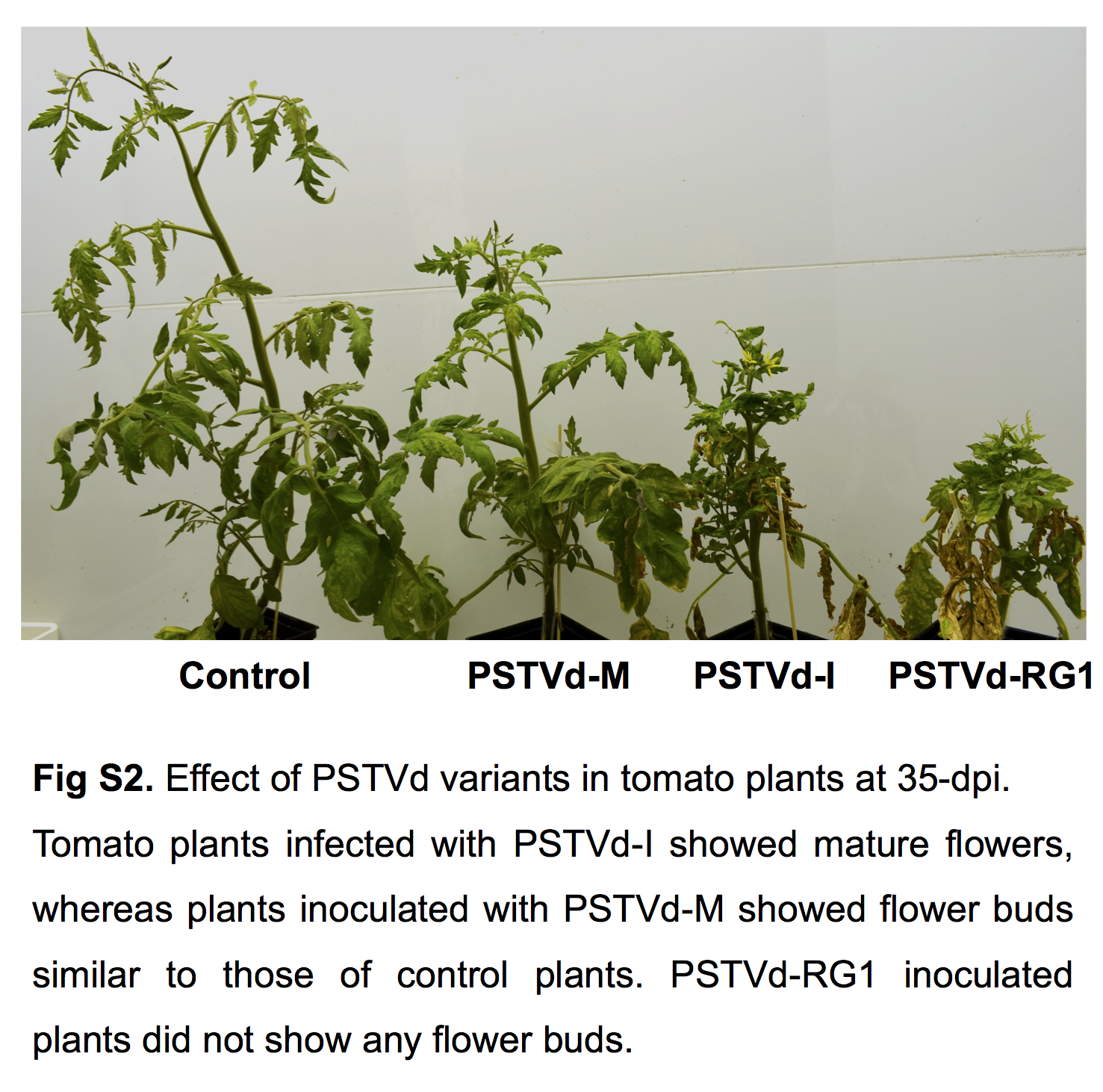

Supplement: Supplementary file 1 — Fig S1 Effect of potato spindle tuber viroid (PSTVd) variants in tomato plants at 35 days post‐infection (dpi). [file MPP-19-2446-s001.tiff]
