# Supplementary material for: Viroid‐derived small RNA induces early flowering in tomato plants by RNA silencing
Source: Mol Plant Pathol. 2018 Sep 28;19(11):2446–58. doi: 10.1111/mpp.12721 (PMC6637976; doi:10.1111/mpp.12721)
Supplement: Supplementary file 2 — Table S1 Primers used in this study. [file MPP-19-2446-s002.docx]

**Table S1. Primers used in this study.**

| Experiment | Target gene | Forward primer (5’ to 3’) | Reverse primer (5’ to 3’) | construct name |
| --- | --- | --- | --- | --- |
| VIGS | FRL3 VIGS | TCTCTAGA CTCTCTGGTTCTGGATTCGC | GCGGATCC GGAAACTGCTCTGTCAGCTC | pTRV:FRL3 |
| qPCR | FRL3 | CCATTACCAGGCTCCTTACC | CCCACACGAGCAAATTAAGC | TGTTCTGTGGAACCAACTGTT |
|  | Potato spindle tuber viroid (PSTVd)^a^ | GCCCCCTTTGCGCTGT | AAGCGGTTCTCGGGAGCTT |  |
|  | Ubiquitin-conjugating  enzyme (UBC)^b^ | GCAATCTTCTTCGATCCGGT | GCTACAGAACACCAAGCAGA |  |
|  | Transducing/WD40  repeat family  protein^b^ | ATAAGCTCCCTGGACACAC | CCTCACCTTCTTCTCAAATCTC |  |
|  | ARF-like GTPase  family protein  (ASAR1)^b^ | GGAGGTGTTTATGTGCAGTATT | CCAGACGGGAAAAAATAGTTGT |  |
| 5’ RLM RACE | 5’ RNA adapter | GUUCAGAGUUCUACAGUCCGACGAUC^C^ |  |  |
|  | FRL3 I PCR | AATGATACGGCGACCACCGACAGGTTCAGAGTTCTACAGTCCGA^C^ | GCTGCCAGGTTTTTACGGTTG |  |
|  | FRL3 nested PCR | CCGACAGGTTCAGAGTTCTAC^C^ | CTCTGGATAAGACTTAACTTC |  |
| vd-sRNA:VIGS | amiR:PDS* | TCTCTAGACTGTAGCAGCAGCAGTCAACATAGACAGATAGGGGCCAGGAGATTCAGTTTGAA | GAGGTACCGCCTAGCAGCAGGAATCAACATAGACTGATTGGGGCAGAGAGGCAAAAGTGAA | pCV-amiR:PDS |
|  | amiR:FRL3* | TCTCTAGACTGTAGCAGCAGCAGCGCAGTTGGTTGCTCCGAACTCAGGAGATTCAGTTTGAA | GAGGTACCGCCTAGCAGCAGGAACGCAGTTGGTTCCTCGGAACTAGAGAGGCAAAAGTGAA | pCV-amiR:FRL3 |
| Gel blot | FRL3 probe | CCTGCAGTGGAAGCAACTTG | GAATCTCCTCCCTGAGTGCTG |  |
|  | 5S probe | AGGGGGTCACCCATCCTAGTACTACTCTCGCCCAAGCACGCTTAAC |  |  |

^a^Boonham et al., 2004 (1)

^b^Dekkers et al., 2012 (2)

^C^Adkar-Purushothama et al., 2015 (3)

*osa-MIR528 was used as template to generate amiR:PDS and amiR:FRL3

*Bam*HI (ggatcc), *Xba*I (tctaga) and, *Kpn*I (ggatcc) sites were included in the PCR primers for the cloning of the amplified products into the pTRV2 vector.

*Xba*I (tctaga) and KpnI (ggatcc) sites were included in the PCR primers for the cloning of the amplified products into the pCV-A vector.

The underlined letters in the primers indicate the amiRNA sequence of vd-sRNA.

**References**

**Adkar-Purushothama CR, Brosseau C, Giguère T, Sano T, Moffett P, Perreault J-P**. 2015. Small RNA Derived from the Virulence Modulating Region of the Potato spindle tuber viroid Silences callose synthase Genes of Tomato Plants. The Plant cell **27**, 2178–94.

**Boonham N, Pérez LG, Mendez M., Peralta EL, Blockley A, Walsh K, Barker I, Mumford R.** 2004. Development of a real-time RT-PCR assay for the detection of Potato spindle tuber viroid. Journal of Virological Methods **116**, 139–146.

**Dekkers BJW, Willems L, Bassel GW, Van Bolderen-Veldkamp RPM, Ligterink W, Hilhorst HWM, Bentsink L.** 2012. Identification of reference genes for RT-qPCR expression analysis in arabidopsis and tomato seeds. Plant Cell Physiol 53:28–37.
